# Recent progress in [^11^C]carbon dioxide ([^11^C]CO_2_) and [^11^C]carbon monoxide ([^11^C]CO) chemistry

**DOI:** 10.1002/jlcr.3596

**Published:** 2018-02-05

**Authors:** Carlotta Taddei, Antony D. Gee

**Affiliations:** ^1^ School of Biomedical Engineering and Imaging Sciences King's College London London UK

**Keywords:** [^11^C]CO, [^11^C]CO_2_, ^11^C‐carbonylation, ^11^C‐carboxylation, ^11^C‐labelling, carbon‐11, CO‐releasing molecules, PET

## Abstract

[^11^C]Carbon dioxide ([^11^C]CO_2_) and [^11^C]carbon monoxide ([^11^C]CO) are 2 attractive precursors for labelling the carbonyl position (C═O) in a vast range of functionalised molecules (eg, ureas, amides, and carboxylic acids). The development of radiosynthetic methods to produce functionalised ^11^C‐labelled compounds is required to enhance the radiotracers available for positron emission tomography, molecular, and medical imaging applications. Following a brief summary of secondary ^11^C‐precursor production and uses, the review focuses on recent progress with direct ^11^C‐carboxylation routes with [^11^C]CO_2_ and ^11^C‐carbonylation with [^11^C]CO. Novel approaches to generate [^11^C]CO using CO‐releasing molecules (CO‐RMs), such as silacarboxylic acids and disilanes, applied to radiochemistry are described and compared with standard [^11^C]CO production methods. These innovative [^11^C]CO synthesis strategies represent efficient and reliable [^11^C]CO production processes, enabling the widespread use of [^11^C]CO chemistry within the wider radiochemistry community.

## 1 INTRODUCTION

### 1.1 Production and applications

**Scheme 1:**
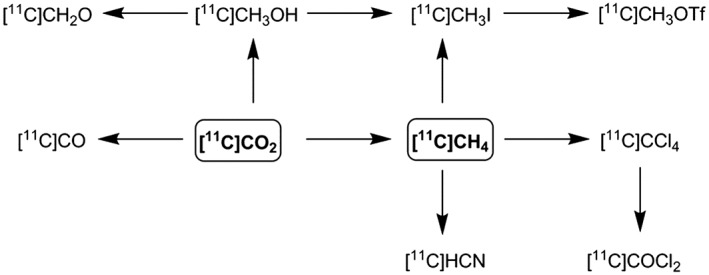
Primary and secondary ^**11**^C‐precursors

Carbon‐11 (^11^C) is an unstable positron‐emitting isotope of carbon with a half‐life of 20.4 minutes. It is generally produced using a cyclotron by the proton bombardment of ^14^N according to the following nuclear reaction: ^14^N(p, α)^11^C. The 2 major primary ^11^C‐precursors used in radiosynthesis are [^11^C]CO_2_ and [^11^C]CH_4_. These are produced in the gas target when the proton bombardment of ^14^N occurs in the presence of traces of oxygen (0.5%–1%) or hydrogen (5%–10%), respectively.^1^ One of the main challenges in ^11^C‐chemistry is the development of rapid, versatile, and reliable methods to integrate these primary ^11^C‐precursors into functionalised molecules.^2^ Despite the low reactivity of [^11^C]CO_2_ and [^11^C]CH_4_, an extensive number of methods have been developed to label functionalised ^11^C‐molecules from these ^11^C‐precursors.^3^, ^4^, ^5^ [^11^C]CO_2_ and [^11^C]CH_4_ can be also transformed into more reactive secondary ^11^C‐precursors, Scheme 1. These, however, often require significant processing times and vary in yields.

### 1.2 [^11^C]methyl iodide, [^11^C]methyl triflate, [^11^C]hydrogen cyanide, [^11^C]phosgene

#### 1.2.1 [^11^C]methyl iodide and [^11^C]methyl triflate

**Scheme 2:**
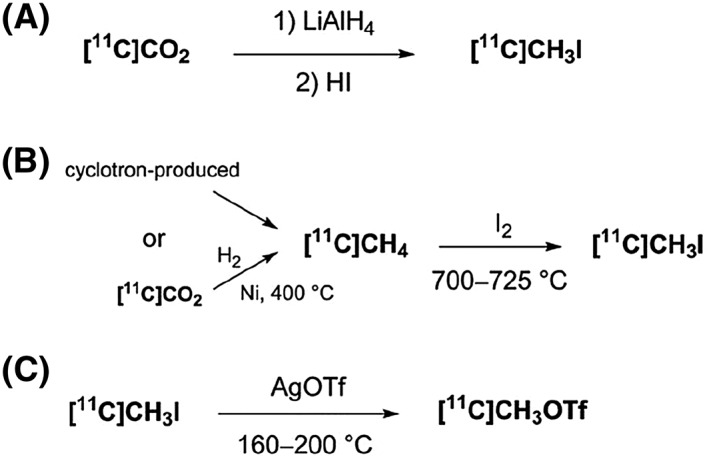
Production of ^**11**^C‐methylating reagents

One of the most widespread ^11^C‐incorporation methodology uses [^11^C]methyl iodide ([^11^C]CH_3_I) as a ^11^C‐methylation reagent. [^11^C]CH_3_I can be generated *via* the “wet” method or the gas‐phase method. The first approach involves the reduction of cyclotron‐produced [^11^C]CO_2_ with LiAlH_4_ followed by reaction with HI, Scheme 2 (A).^6^ The second method is based on the gas‐phase iodination of [^11^C]CH_4_, which can be formed directly from the cyclotron or by reduction of [^11^C]CO_2_ in the presence of hydrogen gas on a nickel support at high temperatures, Scheme 2 (B).^7^, ^8^ The [^11^C]CH_4_ is then exposed to gas‐phase radical iodination using iodine vapour at 700°C to 725°C to yield the desired labelling agent [^11^C]CH_3_I, Scheme 2 (B).

[^11^C]Methyl triflate ([^11^C]CH_3_OTf), another ^11^C‐methylation reagent, is generally prepared by passing gaseous [^11^C]CH_3_I over silver triflate at 160°C to 200°C, Scheme 2 (C).^9^ Due to its higher reactivity than [^11^C]CH_3_I, this labelling agent has recently found increased utilisation.

**Scheme 3:**
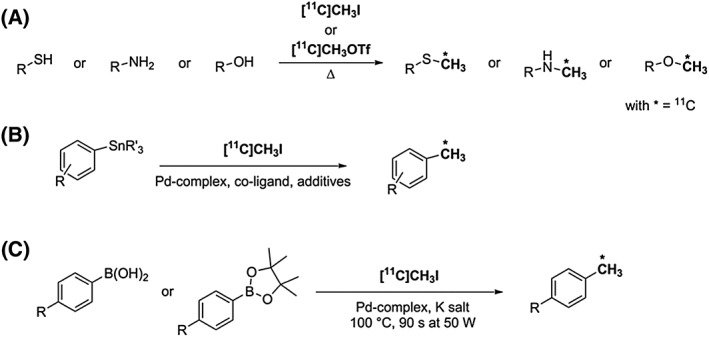
^**11**^C‐methylation reactions: (A) nucleophilic substitution on thiols, amine, and alcohols; (B) Stille cross‐coupling with organostannanes; (C) Suzuki cross‐coupling with boron compounds

^11^C‐Methylation reactions generally involve nucleophilic substitution of [^11^C]CH_3_I or [^11^C]CH_3_OTf with a primary amine, alcohol, or thiol group to form the corresponding secondary amine, ether or thioether, Scheme 3 (A). This approach requires the trapping of the ^11^C‐methylation reagents in a solution of the precursor followed by heating for a short period of time. Due to its simplicity, ^11^C‐methylation is widely used for research and clinical production of functionalised ^11^C‐tracers as extensively reviewed in the literature.^2^, ^4^, ^5^, ^10^, ^11^, ^12^, ^13^, ^14^, ^15^, ^16^, ^17^, ^18^ The recent development of “loop” chemistry has enabled technical and yield improvements in ^11^C‐methylation reactions.^2^

“Loop” ^11^C‐methylation involves depositing a solution of the reagents in a thin film on the inside of an HPLC loop. The passage of [^11^C]CH_3_I or [^11^C]CH_3_OTf through this loop produces the methylated ^11^C‐product.^19^ This approach allows a high reactive surface area, minimal technical handling, and simplified ^11^C‐product purification leading to improved ^11^C‐methylation reaction yields.

^11^C‐Methylation has also been applied in palladium‐mediated cross‐coupling reactions for ^11^C―C bond formation to radiolabel molecules of interest with ^11^C in specific positions. Good functional group tolerance has been shown using organostannanes as precursors in Stille cross‐coupling reactions, Scheme 3 (B).^20^, ^21^ [^11^C]CH_3_I is typically trapped in a solution containing a Pd‐complex and a co‐ligand. This mixture is then transferred in a vial containing the organostannane and heated for a few minutes (2–5 minutes). Despite the broad functional group compatibility, toxic trace amounts of stannanes are difficult to remove completely from the reaction mixture and may raise concerns about this methodology for in vivo applications.

The Suzuki cross‐coupling reaction using boronic acids and boronic esters as precursors is an alternative route to ^11^C―C bond formation which avoids concerns about using organostannane reagents, Scheme 3 (C).^20^, ^22^, ^23^ In analogy to the Stille coupling, [^11^C]CH_3_I is added to a solution containing a Pd‐complex, the boronic acid (or boronic ester), and a potassium salt. This mixture is then heated (eg, by microwave [MW] activation), and the reaction is quenched with water, Scheme 3 (C).

#### 1.2.2 [^11^C]hydrogen cyanide

**Scheme 4:**
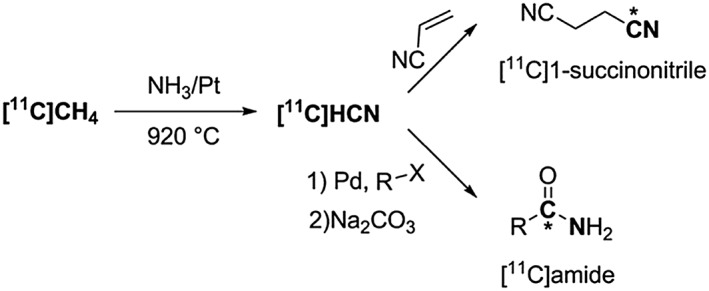
Production of [^**11**^C]HCN and its use in ^**11**^C‐cyanation reactions

[^11^C]Hydrogen cyanide ([^11^C]HCN) is another useful secondary ^11^C‐precursor for the synthesis of functionalised ^11^C‐tracers.^24^, ^25^, ^26^, ^27^, ^28^ It is commonly produced by the conversion of [^11^C]CH_4_ in the presence of NH_3_ over platinum at high temperatures, Scheme 4.^29^ [^11^C]HCN can be used for ^11^C‐cyanation reactions, such as for the production of [^11^C]1‐succinonitrile^2^, ^30^ or converted to other functional groups, such as [^11^C]amides,^2^, ^31^ Scheme 4.

#### 1.2.3 [^11^C]phosgene

**Scheme 5:**
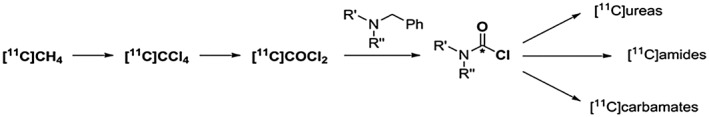
Production of [^**11**^C]COCl_2_ and subsequent synthesis of [^**11**^C]ureas, [^**11**^C]carbamates, and [^**11**^C]amides

[^11^C]Phosgene ([^11^C]COCl_2_) is usually produced by the chlorination of [^11^C]CH_4_ to [^11^C]CCl_4_ followed by oxidation to [^11^C]COCl_2_.^32^ Thanks to its high reactivity, [^11^C]COCl_2_ can be utilised for the synthesis of functionalised [^11^C]ureas, [^11^C]carbamates, and [^11^C]amides *via* formation of the corresponding [^11^C]carbamoyl chlorides, Scheme 5.^33^ However, the production of [^11^C]COCl_2_ has been found to lack reliability and reproducibility at some radiochemistry sites, limiting its widespread use in ^11^C‐chemistry.^34^

### 1.3 Direct ^11^C‐carboxylation

**Scheme 6:**
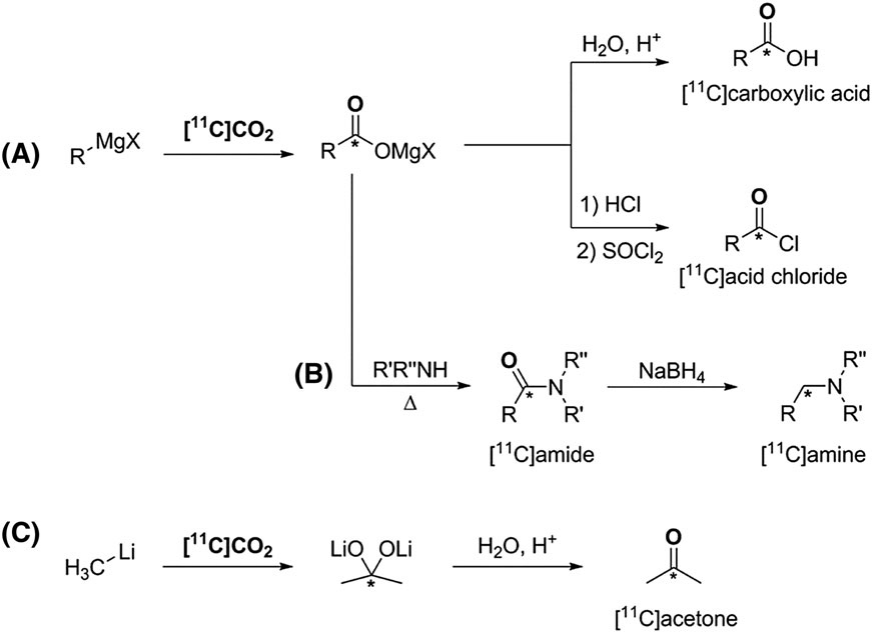
(A) and (B) [^**11**^C]CO_2_ fixation using Grignard regents; (C) [^**11**^C]CO_2_ incorporation into organolithium reagents

Despite its low reactivity and solubility in organic solvents, the direct incorporation of cyclotron‐produced [^11^C]CO_2_ is of great interest because, in principle, rapid synthesis times might be achieved with a reduced number of reaction steps and technical processing. Several methodologies have been developed to access a vast range of ^11^C‐tracers, including [^11^C]carboxylic acids, [^11^C]esters, [^11^C]amides, [^11^C]amines, [^11^C]ureas, [^11^C]carbamates, and [^11^C]acid chlorides.^2^, ^3^, ^35^, ^36^, ^37^, ^38^, ^39^, ^40^, ^41^, ^42^ The direct carboxylation of Grignard reagents with [^11^C]CO_2_ enables the rapid synthesis of [^11^C]carboxylic acids and [^11^C]acid chlorides, Scheme 6 (A). These have been shown to be useful ^11^C‐reagents for the synthesis of functionalised radiopharmaceuticals, such as [^11^C]WAY 100365.^43^ The produced ^11^C‐carboxylate intermediates can also be utilised to yield the corresponding [^11^C]amides from the reaction with primary and secondary amines, Scheme 6 (B). Furthermore, the synthesised [^11^C]amides can be subsequently reduced yielding the corresponding [^11^C]amines, Scheme 6 (B).^3^

Using a similar approach, organolithium reagents readily react with [^11^C]CO_2_ producing the corresponding [^11^C]ketones. For example, [^11^C]acetone is obtained from the coupling of [^11^C]CO_2_ with methyllithium followed by hydrolysis, Scheme 6 (C).^3^ [^11^C]Acetone has itself been utilised as a useful labelling intermediate in ^11^C‐chemistry.^44^, ^45^, ^46^

Grignard and organolithium reagents are often used in ^11^C‐chemistry due to their great reactivity as nucleophiles for [^11^C]CO_2_. However, as a consequence of their reactivity, these reagents do not have wide functional group compatibility and readily react with atmospheric CO_2_ lowering the molar activity (A_m_) of the final ^11^C‐tracer. This aspect restricts the functionalised ^11^C‐molecules achievable using this methodology. In addition, the required careful handling under inert atmosphere limits the routine applicability of these reagents.^3^

**Scheme 7:**
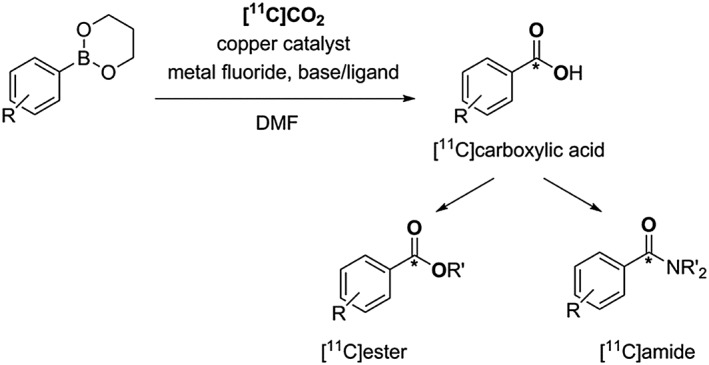
[^**11**^C]CO_2_ incorporation into benzyl boronic esters

Other carboxylation methods using [^11^C]CO_2_ have been developed in order to overcome the limitations of Grignard and organolithium reagents. An example is the copper‐catalysed incorporation of [^11^C]CO_2_ into the more stable and less moisture sensitive boronic esters yielding functionalised [^11^C]carboxylic acids, Scheme 7.^35^, ^47^ These can be subsequently transformed to [^11^C]esters or [^11^C]amides, Scheme 7.^35^ However, 1 drawback of this methodology relies on its restriction to benzyl and unsaturated aliphatic boronic esters.

**Scheme 8:**
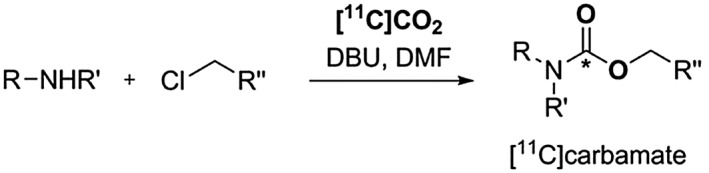
Direct fixation of [^**11**^C]CO_2_ yielding [^**11**^C]carbamates

As discussed earlier, 2 main challenges in the trapping of [^11^C]CO_2_ are its solubility in the reaction media and its low reactivity towards nucleophiles. The advent of fixation agents, such as 1,8‐diazabicyclo[5.4.0]undec‐7‐ene (DBU) and 2‐*tert*‐butylimino‐2‐diethylamino‐1,3‐dimethylperhydro‐1,3,2‐diazaphosphorine (BEMP), has overcome this issue and has enabled the further development of new synthesis methodologies for functionalised ^11^C‐tracers, such as [^11^C]carbamates and [^11^C]ureas.^36^, ^37^, ^38^, ^39^, ^48^ An example is the 1‐pot synthesis of a wide range of [^11^C]carbamate esters under mild reaction conditions utilising [^11^C]CO_2_ and DBU as a trapping reagent, Scheme 8.^38^, ^39^

**Scheme 9:**
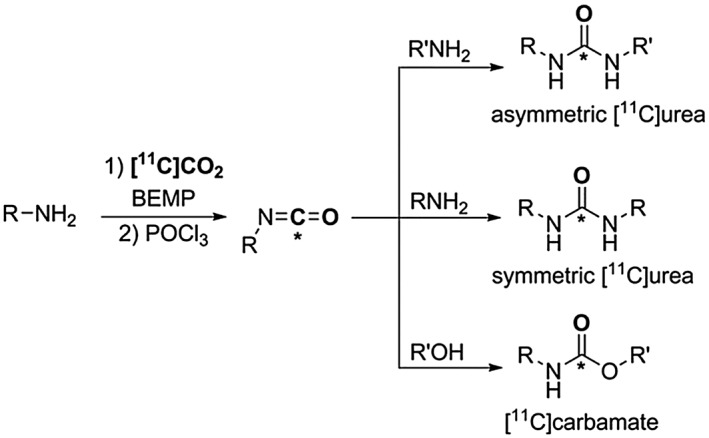
Synthesis of [^**11**^C]ureas or [^**11**^C]carbamates from [^**11**^C]isocyanates

It has been found that by stoichiometric control of the reagents, the [^11^C]carbamate salts can be dehydrated to [^11^C]isocyanates and transformed into [^11^C]ureas or [^11^C]carbamates, Scheme 9.^48^ Despite the broad applicability of this methodology, low yields were obtained for the more unreactive aromatic amines.

**Scheme 10:**
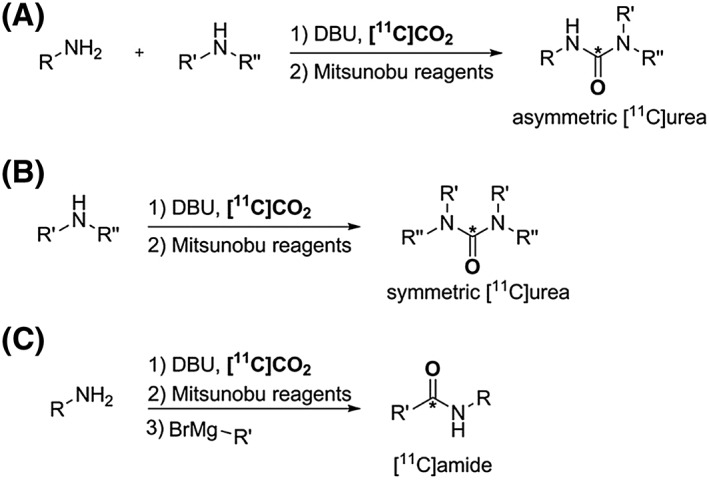
(A) and (B) Synthesis of asymmetrical and symmetrical [^**11**^C]ureas *via* Mitsunobu reaction. (C) Synthesis of [^**11**^C]amides *via* Mitsunobu reaction

Two novel methodologies based on [^11^C]CO_2_ trapping in the presence of BEMP and subsequent addition of Mitsunobu reagents have been developed, Scheme 10 (A and B) to expand the range of functionalised [^11^C]ureas.^36^, ^37^ A similar approach has been also recently discovered for the synthesis of [^11^C]amides *via* rapid addition of Grignard regents after Mitsunobu reaction, Scheme 10 (C).^49^ These direct [^11^C]CO_2_ fixation methodologies are attractive alternatives for the synthesis of functionalised [^11^C]ureas and [^11^C]amides compared with the [^11^C]COCl_2_‐based methods.

**Scheme 11:**
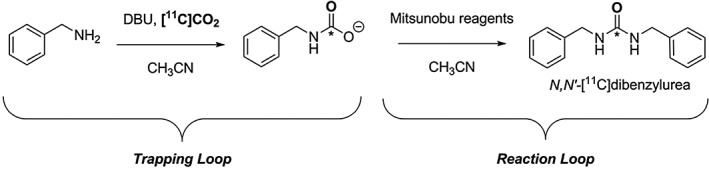
[^**11**^C]CO_2_ trapping loop combined with a reaction loop for the Mitsunobu reaction yielding *N,N′*‐[^**11**^C]dibenzylurea presented by Downey et al

Based on the potential of Mitsunobu reactions, a continuous‐flow loop setup for [^11^C]CO_2_ trapping and [^11^C]ureas synthesis has been recently presented by Downey et al.^50^, ^51^ This work demonstrated the rapid and efficient [^11^C]CO_2_ trapping in DBU/amine solutions (average of 78%) at a high delivery flow rate (70 mL/min) within a low volume polymer loop (150 μL). This [^11^C]CO_2_ trapping system was integrated into a continuous‐flow ^11^C‐labelling of a model symmetric urea, *N,N′‐*[^11^C]dibenzylurea *via* Mitsunobu reaction, Scheme 11. *N,N′‐*[^11^C]Dibenzylurea was obtained in high decay‐corrected radiochemical yield (RCY) of up to 72% and crude radiochemical purity (RCP) of up to 83% under ambient temperature and pressure within short synthesis time (<3 minutes from end of delivery [EOD]).^51^

A very similar approach has been recently reported by Dahl et al to produce a diverse range of compounds, including [^11^C]carbamates, [^11^C]oxazolidinones, and [^11^C]ureas in good decay‐corrected RCYs (18%–50%) and high isolated RCPs (>99%).^52^ This work together with the results presented by Downey et al demonstrates the utility of a simple and efficient “in‐loop” [^11^C]CO_2_ trapping method allowing the reliable production of a diverse array of ^11^C‐products with minimal loss in radioactivity. This approach might be useful in a routine environment for positron emission tomography (PET) tracer development.

## 2 [^11^C]CARBON MONOXIDE ([^11^C]CO)

### 2.1 Production: Oven‐based method

**Scheme 12:**
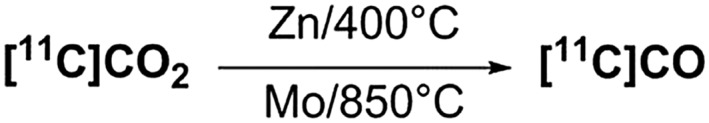
Reduction of [^**11**^C]CO_2_ to [^**11**^C]CO on a metal surface

[^11^C]Carbon monoxide ([^11^C]CO) was one of the first ^11^C‐tracers used for blood volume measurements in humans.^53^ [^11^C]CO is generally produced by the gas‐phase reduction of cyclotron‐produced [^11^C]CO_2_ on a metal surface (zinc or molybdenum) placed in a heated quartz tube at high temperatures, Scheme 12.^54^, ^55^, ^56^, ^57^

One of the first developed [^11^C]CO synthesis methodologies was the reduction of [^11^C]CO_2_ to [^11^C]CO on a zinc heated column (400°C) followed by concentration of the produced [^11^C]CO on a silica column. This method produced low [^11^C]CO yields and low trapping efficiency (~10%) for 2 main reasons:

1. the high flow rate used (100–200 mL/min) to deliver [^11^C]CO to the reaction vial,
2. the re‐oxidation of [^11^C]CO to [^11^C]CO_2_ upon heating of the silica column.^55^

These factors triggered the development of improved [^11^C]CO gas handling systems.

The pre‐concentration of [^11^C]CO_2_ prior reduction and the introduction of a [^11^C]CO recirculation unit allowed [^11^C]CO yields of up to 70%.^55^, ^57^ Furthermore, reduced delivery flow rates (20–30 mL/min) improved the [^11^C]CO trapping efficiency in organic solvents.^55^

A further development in [^11^C]CO chemistry was the introduction of high pressure micro‐autoclaves and “loop” synthesis systems. These assured an efficient [^11^C]CO trapping in the reaction mixture thanks to a very low gas‐phase volume and a higher reaction efficiency due to the greater reactive surface area and elevated pressures.^58^

Methods for the reduction of [^11^C]CO_2_ using zinc ovens often suffer from the degradation of the metal surface by formation of zinc oxides over a few [^11^C]CO production cycles. Zinc columns require frequent changes, cleaning, and careful pre‐purification of the [^11^C]CO_2_ in order to assure reproducible [^11^C]CO yields.^54^, ^56^, ^59^ In addition, the melting point of zinc (420°C) is close to the temperature required for the [^11^C]CO_2_ reduction to occur (400°C). Therefore, the inadvertent overheating of the zinc column during the process is a risk to the robustness of this method.^56^

The use of molybdenum as a reducing metal in high‐pressure systems has recently shown more reproducible [^11^C]CO yields compared with the zinc method.^54^ Molybdenum is known to readily react with [^11^C]CO_2_ to form [^11^C]CO and molybdenum oxide with a maximum efficiency at 850°C.^56^ The latter has also shown reducing properties towards [^11^C]CO_2_ yielding [^11^C]CO, which might improve the performance of the system and avoid repeated maintenance.^56^ This methodology enables the production of [^11^C]CO in yields of up to 70% over several production cycles.^54^ In addition, the high melting point of this metal (>>850°C) avoids the risk of catalyst melting during the conversion process.

Zinc and molybdenum ovens are used as the standard method for generating [^11^C]CO from [^11^C]CO_2_. However, the need of dedicated infrastructure for these oven‐based methods often limits the use of [^11^C]CO chemistry within the wider radiochemistry community.

An innovative [^11^C]CO production methodology has been recently developed under mild reaction conditions *via* electrochemical conversion of [^11^C]CO_2_ to [^11^C]CO catalysed by nickel and zinc complexes.^60^ Despite the appealing features of this method, only low [^11^C]CO yields were achieved (~10%). Therefore, novel [^11^C]CO synthesis methodologies based on simple laboratory setups leading to comparable [^11^C]CO yields to the standard oven‐based methods are required to enhance the availability of [^11^C]CO for ^11^C‐tracer development.

### 2.2 ^11^C‐Carbonylation reactions

**Scheme 13:**
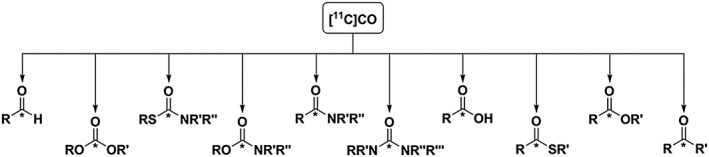
Potential ^**11**^C‐labelled compounds using [^**11**^C]CO

Because of the ubiquity of the C═O functional group in many biologically active molecules, the chemical versatility of CO and the potential of palladium‐promoted carbonylation cross‐coupling reactions have made [^11^C]CO an attractive tool for the development of ^11^C‐chemistry methodologies. To date, [^11^C]CO has been used for direct ^11^C‐carbonylation reactions producing a vast range of ^11^C‐compounds, such as [^11^C]amides, [^11^C]ureas, [^11^C]carboxylic acids, and [^11^C]esters, Scheme 13.^34^, ^55^, ^57^, ^59^, ^61^, ^62^, ^63^, ^64^, ^65^, ^66^, ^67^, ^68^, ^69^, ^70^, ^71^, ^72^ Compared with traditional chemical methods, a major challenge in radiochemistry is the reaction stoichiometry, because in radiochemistry the amount of ^11^C produced is generally in the nano‐picomolar range (10^−9^–10^−12^ mol). Even “low levels” of impurities in the reagents and solvents used may be present in excess compared with the radiolabelled starting material. As a result, reactions working on a traditional chemistry scale can fail when translated to tracer radiochemistry, affecting the outcome of the radiolabelling reactions employed.

### 2.3 Mechanism of ^11^C‐carbonylation with [^11^C]CO

**Scheme 14:**
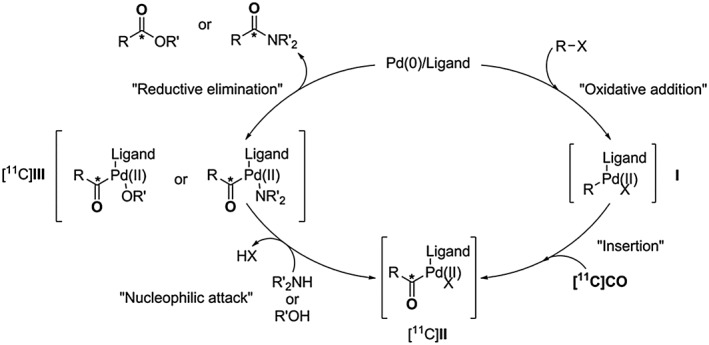
^**11**^C‐Carbonylation reaction mechanism leading [^**11**^C]amides and [^**11**^C]esters

In radiochemistry, [^11^C]CO is typically delivered in a stream of nitrogen, helium, or xenon gas into a vial or a micro reactor containing carbonylation reagents: a palladium ligand complex, an organic halide and an amine or an alcohol. The reaction mechanism starts with the oxidation of the palladium/ligand complex due to addition to the organic halide, Scheme 14. It proceeds with the [^11^C]CO insertion into complex **I** yielding intermediate [^11^C]**II**. Subsequent nucleophilic attack of an amine or an alcohol to the palladium centre gives intermediate [^11^C]**III** with elimination of the corresponding halogen acid. The subsequent reductive elimination of the palladium/ligand complex from intermediate [^11^C]**III** produces a [*carbonyl‐* ^11^C]amide or a [*carbonyl‐* ^11^C]ester with regeneration of the reduced palladium/ligand complex, Scheme 14. Low pressure Pd‐mediated and Rh‐mediated ^11^C‐aminocarbonylations have shown to be adaptable to a broad range of applications, such as the production of [^11^C]amides and [^11^C]ureas.^59^ Because of the high solubility of xenon in organic solvents, the use of this gas as a [^11^C]CO delivery vector enables the transfer of [^11^C]CO into small volumes without a build‐up of pressure.^59^ This methodology is appealing as it does not require additional CO trapping reagents to efficiently trap [^11^C]CO in the carbonylation reaction vessel. Other work has shown the application of a photoinduced radical‐mediated ^11^C‐alkoxycarbonylation reaction to generate [^11^C]esters. This approach affords functionalised aliphatic [^11^C]esters from primary, secondary, and tertiary alkyl iodides.^73^ However, it requires specialised equipment for the photoinduction of the ^11^C‐carbonylation reaction.

## 3 CO‐RELEASING MOLECULES (CO‐RMS)

**Scheme 15:**
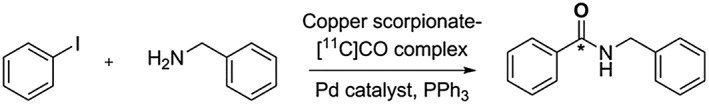
Copper scorpionate‐[^**11**^C]CO complex and in situ ^**11**^C‐carbonylation reaction

Carbon monoxide‐releasing molecules (CO‐RMs) are compounds able to release carbon monoxide under specific conditions. Past studies have shown the application of CO‐RMs in medicine as therapeutic agents^74^, ^75^ and in synthetic chemistry as CO trapping‐releasing agents.^69^, ^76^, ^77^, ^78^, ^79^, ^80^, ^81^, ^82^, ^83^ The synthesis of metal carbonyl complexes, such as rhuthenium‐CO and copper‐CO complexes, and their application as in situ CO‐releasing molecules have rapidly increased.^69^, ^84^, ^85^, ^86^, ^87^, ^88^ These complexes are able to release CO under physiological conditions^84^ or by addition of a competing ligand.^69^ The latter approach was successfully applied to ^11^C‐chemistry using a copper(I) tris(pyrazolyl)borate ligand (so‐called “scorpionate” ligand), Scheme 15. This complex efficiently trapped [^11^C]CO, and by addition of PPh_3_ as a competing ligand, [^11^C]CO was released and subsequently utilised for in situ ^11^C‐carbonylation reactions yielding functionalised [^11^C]amides, Scheme 15.^69^

**Scheme 16:**
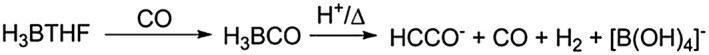
Borocarbonates complexes as CO‐RMs

Using a similar approach, recent non‐radiochemical studies have focused on in situ CO production mediated by molecules able to release CO upon heating. For example, boranocarbonates have demonstrated the ability to release CO during thermolysis, Scheme 16. These compounds have been successfully applied in radiochemistry for the production of ^99m^Tc‐complexes used in radiopharmaceutical applications.^89^ In addition, THF–BH_3_ has been implemented in ^11^C‐chemistry due to its ability to readily retain [^11^C]CO *via* the formation of solvent‐soluble adducts, such as BH_3_‐[^11^C]CO (b.p. −64°C). [^11^C]CO was trapped in organic solvents at ambient temperature and pressure in high efficiency (>95%) and utilised in subsequent palladium‐mediated ^11^C‐carbonylation reactions.^90^

**Scheme 17:**
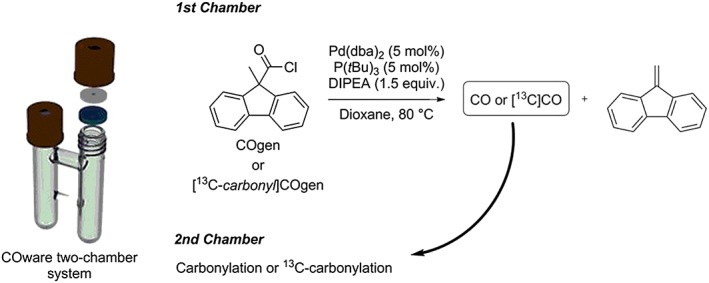
COware 2‐chamber system^92^; COgen^92^ (first chamber) for ex situ carbonylation reactions (second chamber)

Many other CO production methodologies utilising aldehydes, carbamoylsilane, carbamoylstannanes, formic acid, and its derivatives have been developed and applied to the synthesis of carbonyl functionalised molecules.^79^, ^80^, ^91^ A recent work demonstrated the ability of 9‐methyl‐9H‐fluorene‐9‐carbonyl chloride (named “COgen” upon commercialisation) to release CO *via* a palladium‐catalysed decarbonylation reaction performed at 80°C, Scheme 17.^93^, ^94^ The combination of this CO‐releasing process with a CO‐consuming reaction in an isolated 2‐chamber system enabled a high trapping of the produced CO. This methodology was also successfully applied to ^13^C‐chemistry for the labelling of aryl amides with [*carbonyl‐* ^13^C]COgen.^93^

## 4 NOVEL [^11^C]CO PRODUCTION METHODOLOGIES

### 4.1 Silacarboxylic acids as CO‐RMs

Other examples of useful CO‐RMs are silacarboxylic acids and disilanes.^76^, ^78^ These have been recently used as in‐situ CO sources for ex‐situ transition‐metal catalysed carbonylation reactions.^76^, ^77^, ^78^, ^95^

**Scheme 18:**
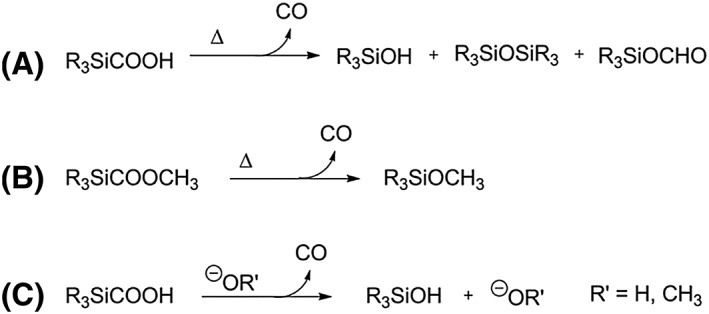
(A) and (B) Thermolysis of silacarboxylic acids and silacarboxylate esters; (C) base‐catalysed CO elimination of silacarboxylic acids

Past works have shown that silacarboxylic acids degrade upon heating (150°C–200°C) with elimination of CO and formation of the corresponding silanol, disiloxane, and the isomeric silyl formate, Scheme 18 (A).^96^, ^97^ Subsequent studies demonstrated that silacarboxylate esters undergo degradation in a similar manner, Scheme 18 (B).^98^ In addition, silacarboxylic acids have shown to lead the corresponding silanol derivative with production of CO in the presence of a base (eg, NaOH), Scheme 18 (C).^96^, ^99^

**Scheme 19:**
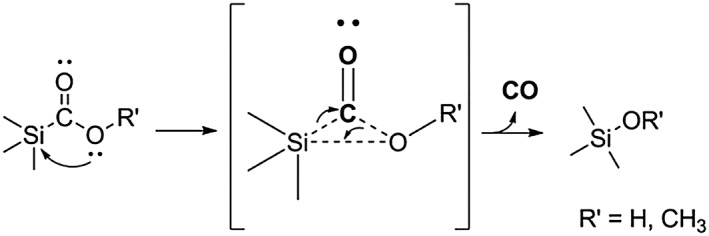
1,2‐Brook rearrangement of silacarboxylate derivatives

The degradation of these compounds was hypothesised to proceed through the attack of a lone pair of electrons of the oxygen atom of the OR′ group to the silicon atom accompanied by elimination of the carbonyl group as CO, Scheme 19. This internal rearrangement was called the 1,2‐Brook rearrangement due the intensive studies on these compounds performed by Brook and co‐workers.^100^ Organosilicon compounds have since found an extensive use in synthetic chemistry, such as in tandem bond formation strategies.^101^, ^102^, ^103^ A similar chemical behaviour has been observed for the same group's elements of silicon, such as germanium.^96^, ^104^

The ability of silacarboxylic acids to release CO under certain conditions and the high fluorophilicity of silicon inspired the exploration of fluoride sources as activators to trigger the release of CO from this class of compounds.^76^ Friis and co‐workers investigated different reaction conditions, such as temperature, reaction time, type of solvent, and activator on a number of silacarboxylic acids. Their results showed Ph_2_MeSiCOOH as yielding the most rapid decarbonylation with production of CO using KF as an activator in dioxane. These reaction conditions were successfully applied in different Pd‐catalysed carbonylation reactions in a 2‐chamber system yielding the corresponding carbonylation product.^76^

The relevance of this CO chemical methodology relies on:

1. the production of a controlled amount of CO using easy‐to‐handle reagents,
2. no need of special infrastructure in laboratories (eg, CO gas cylinder and CO gas detectors),
3. absence of a transition‐metal catalyst,
4. release of CO at ambient temperature.

The 2 latter features distinguish silacarboxylic acids from the previous presented CO‐production methodologies (eg, COgen and boranocarbonates) and made this class of compounds an attractive target for ^11^C‐chemistry application.

### 4.2 Disilanes as CO_2_ to CO reducing agents

**Scheme 20:**
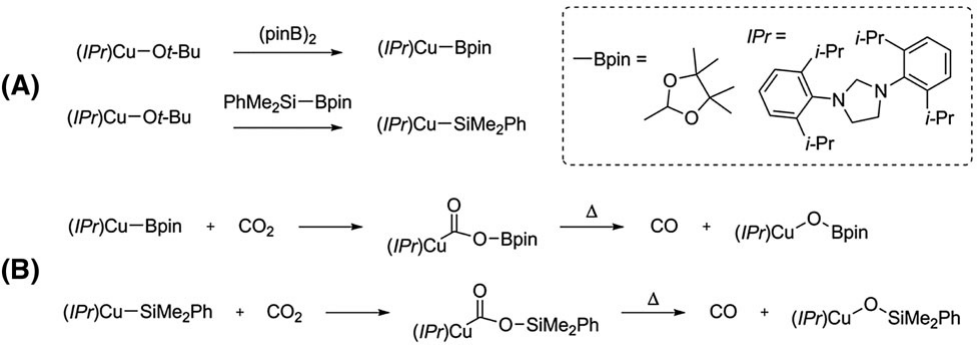
(A) Copper complexes coordinate with diboron and boronsilane reagents; (B) coordination with CO_2_ and release of CO upon thermal decomposition

In parallel with the use of CO‐RMs, others reported the in situ chemical reduction of CO_2_ to CO *via* molecules able to react with CO_2_, remove an oxygen atom from CO_2_, and release CO. An example is the copper complex (*IPr*)Cu―O*t*Bu. This is able to coordinate with diboron compounds^82^ and the structurally related boronsilane compounds^83^ to yield (*IPr*)Cu―Bpin and (*IPr*)Cu―SiMe_2_Ph, respectively. These complexes have shown the ability to coordinate CO_2_ producing the corresponding intermediates *(IPr*)Cu―O_2_CBpin and (*IPr*)Cu―O_2_CSiMe_2_Ph at a low temperatures (−80°C–0°C). Upon thermal decomposition (rt), *(IPr*)Cu―O_2_CBpin and (*IPr*)Cu―O_2_CSiMe_2_Ph release CO with formation of (*IPr*)Cu―OBpin or (*IPr*)Cu―OSiMe_2_Ph, Scheme 20.^82^, ^83^

**Scheme 21:**
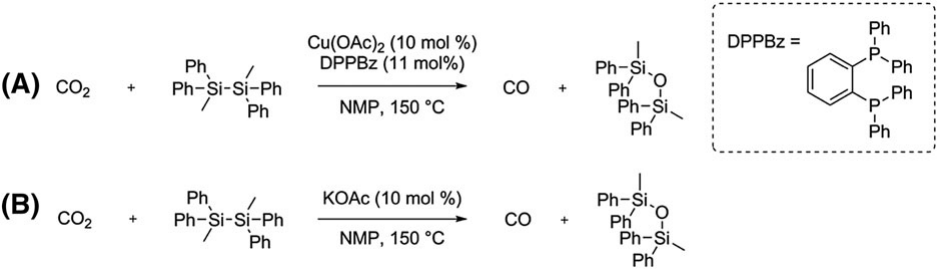
(A) Cu(OAc)_2_/DPPBz complex. (B) KOAc catalysing the CO_2_ to CO transformation *via* (MePh_2_Si)_2_

In order to simplify the catalytic protocol of this CO_2_ to CO reduction, Lescot et al reported that the presence of Cu(OAc)_2_ and the bidentate ligand, DPPBz, with stoichiometric amounts of disilane, (MePh_2_Si)_2_, efficiently reduces CO_2_ to CO with production of the corresponding disiloxane, Scheme 21 (A).^78^ By investigating the influence of different counterions of the copper salt used, they hypothesised that the CO_2_ to CO reduction process could be catalysed in the absence of copper. This was confirmed by the complete conversion of disilane to the corresponding disiloxane with release of CO in the presence of neat KOAc at 150°C, Scheme 21 (B). Further reaction condition optimisation showed that fluoride sources (eg, KF) led to increased reactivity at lower temperatures (80°C). CsF was shown to be an excellent catalyst for the reduction of CO_2_ to CO at ambient temperature with the disilane (MePh_2_Si)_2_.^78^ Investigations on other disilanes showed that disilanes bearing only methyl or phenyl groups were detrimental to the reaction.^78^

Fluoride‐activated disilanes have also been utilised to promote the carboxylation of organic halides under transition‐metal free conditions.^105^ The key aspect of this method is the formation of a silyl anion triggered by fluoride through the Si―Si bond cleavage.

The formation of metal‐free silyl anions in the presence of disilanes and a catalytic amount of tetrabutylammonium fluoride (TBAF) in aprotic solvents (eg, HMPA) has been reported by past studies.^106^ In addition, the generated silyl anions were reacted with aldehydes and 1,3‐dienes to produce the corresponding coupled organosilane products in good yields under extremely mild reaction conditions.^106^, ^107^, ^108^

The ability of disilane species to be activated by hyper‐coordination has become an interesting property for the development of new methodologies in synthetic chemistry and within the ^11^C‐chemistry field.

#### 4.2.1 Bond energies in silicon chemistry

From the presented applications of silacarboxylic acids and disilanes, it is evident that the fluoride anion can promote an intramolecular rearrangement of the Si―C bond or the cleavage of the Si―Si bond. Both routes mediate the formation of Si―O and Si―F bonds.

The formation of the strong Si―F bond can be used as a driving force in silicon chemistry, such as in the cleavage of the weak Si―Si bond (Si―F > Si―O >> Si―C and Si―Si).^109^ In addition, Si―O bond‐dissociation energy >> Si―Si bond‐dissociation energy indicating that the Si―O bond‐dissociation energy can also be utilised as a driving force in silicon chemistry, such as in the 1,2‐Brook rearrangement catalysed by hydroxide and the effect of KOAc on the CO_2_ to CO reduction *via* disilanes.^76^, ^78^ The trend of the bond‐dissociation energies of silicon with halogens is as follows: Si―F >> Si―Cl > Si―Br > Si―I.^109^ Therefore, the substantial fluorophilicity and oxophilicity of silicon in conjunction to its hyper‐coordination properties^110^, ^111^ make organosilicon compounds extremely interesting targets for the development of synthetic and radiosynthetic strategies.

### 4.3 Conversion of [^11^C]CO_2_ to [^11^C]CO *via* [^11^C]silacarboxylic acids

**Scheme 22:**
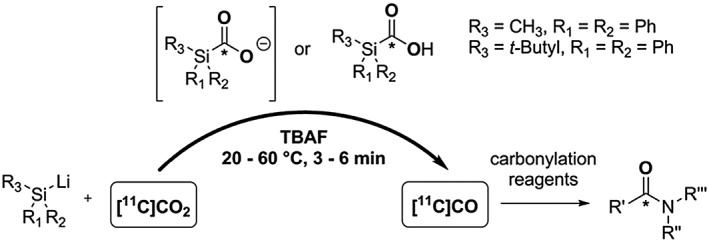
[^**11**^C]CO_2_ to [^**11**^C]CO conversion *via* [^**11**^C]silacarboxylates

An innovative rapid and reliable chemical conversion of [^11^C]CO_2_ to [^11^C]CO mediated by [^11^C]silacarboxylates and [^11^C]silacarboxylic acids triggered by a stoichiometric excess of TBAF has been recently reported by our group and others, Scheme 22.^112^, ^113^, ^114^ This work was inspired by the previously presented non‐radiochemical studies showing silacarboxylic acids as efficient CO‐releasing molecules when in the presence of fluoride.^76^, ^77^

**Scheme 23:**
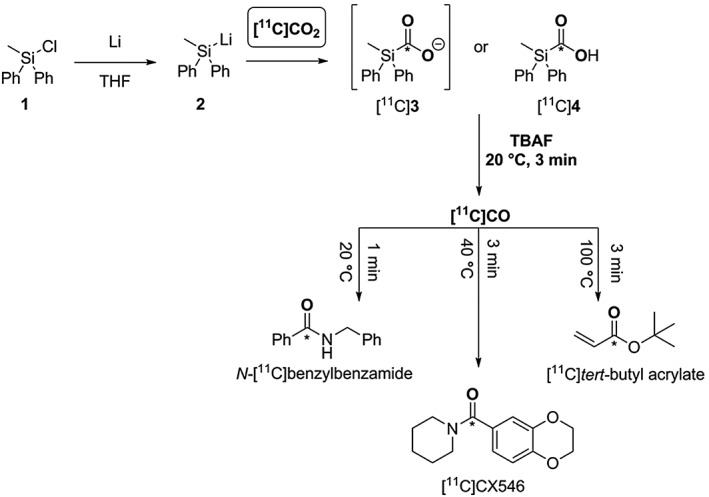
Produced ^**11**^C‐tracers with the [^**11**^C]CO synthesis process *via* [^**11**^C]**3** and [^**11**^C]**4**

In our laboratory, Ph_2_MeSiLi (**2**), synthesised from the corresponding chlorosilane (**1**), was chosen for method development after an initial screening of different silyl lithium derivatives. The corresponding [^11^C]silacarboxylate ([^11^C]**3** and [^11^C]**4**) was obtained in good to high RCY (~40%–80%) by coupling crude **2** with cyclotron‐produced [^11^C]CO_2_. [^11^C]CO production yields ≥50% based on total [^11^C]CO_2_ were obtained either with [^11^C]**3** or [^11^C]**4** within short synthesis time (3 minutes from EOD) and mild reaction conditions (ambient temperature), Scheme 23. Mechanistic investigations revealed that [^11^C]CO yields of 80% ± 20% from [^11^C]**4** could be produced within 3 minutes from EOD at ambient temperature.^114^

The utility of this [^11^C]CO synthesis process was confirmed by radiolabelling functionalised amides and esters, *N‐*[^11^C]benzylbenzamide, [^11^C]CX546 and [^11^C]*tert*‐butyl acrylate,^115^ in good RCY (>30%) and high RCP (>70%) within 6 minutes from EOD, Scheme 23. The automated synthesis system based on a 2‐vial setup using an Eckert and Ziegler Modular Lab apparatus has been successfully tested^112^ yielding *N‐*[^11^C]benzylbenzamide and [^11^C]CX546 in A_m_ of ~60 to 90 GBq/μmol.^116^

This novel [^11^C]CO production methodology is based on a simple labware setup and utilises mild reaction conditions enabling the production of [^11^C]CO in different laboratory configurations without the need for the traditional dedicated [^11^C]CO infrastructure (eg, oven‐based methods). However, this method requires the prior preparation of the silyl lithium precursor and addition of TBAF post [^11^C]CO_2_ delivery, which may be a limiting aspect to its applicability in a routine setting.

### 4.4 Production of [^11^C]CO *via* fluoride‐activated disilanes

Due to the remaining caveats implied in the [^11^C]CO synthesis *via* [^11^C]silacarboxylic acids, our group focused on fluoride‐activated disilanes as [^11^C]CO_2_ reducing agents to develop an improved [^11^C]CO synthesis methodology. This work was inspired by the non‐radiochemical studies showing disilanes as CO_2_ to CO reducing agents when in the presence of a fluoride source.^78^

(MePh_2_Si)_2_ (disilane **a**) was chosen as disilane for method development and reaction optimisation. Various fluoride sources were investigated showing TBAF as the most efficient activator for [^11^C]CO release compared with other fluoride salts. Different solvents were explored revealing THF as the most efficient reaction media for this process. It has been reported that polar aprotic solvents, such as THF, increase the solubility of disilanes and the reactivity of the fluoride anion.^106^, ^117^ 0.1 equiv. of TBAF showed to be optimum for the [^11^C]CO_2_ conversion. No [^11^C]CO production was observed in the absence of TBAF or disilane or the TBAF/disilane complex. No [^11^C]CO production was observed when other TBA salts (eg, TBAB and TABCl) were used instead of TBAF. This demonstrated the relevance of silicon's high fluorophilicity (Si–F >> Si–Br > Si–Cl > Si–I)^109^ in the [^11^C]CO_2_ to [^11^C]CO reduction process. A [^11^C]CO yield of 59% from total cyclotron‐produced [^11^C]CO_2_ was achieved by decreasing the [^11^C]CO_2_ delivery flow rate from 60 mL/min to 10 mL/min. Various disilanes were investigated demonstrating that by using (Me_2_PhSi)_2_ (disilane **d**), TBAF (0.1 equiv.), and THF, [^11^C]CO_2_ was converted to [^11^C]CO in RCYs of 74 ± 6% within 10 minutes from end of bombardment (EOB) under mild reaction conditions (ambient temperature) and at flow rate of 10 mL/min.^118^

**Scheme 24:**
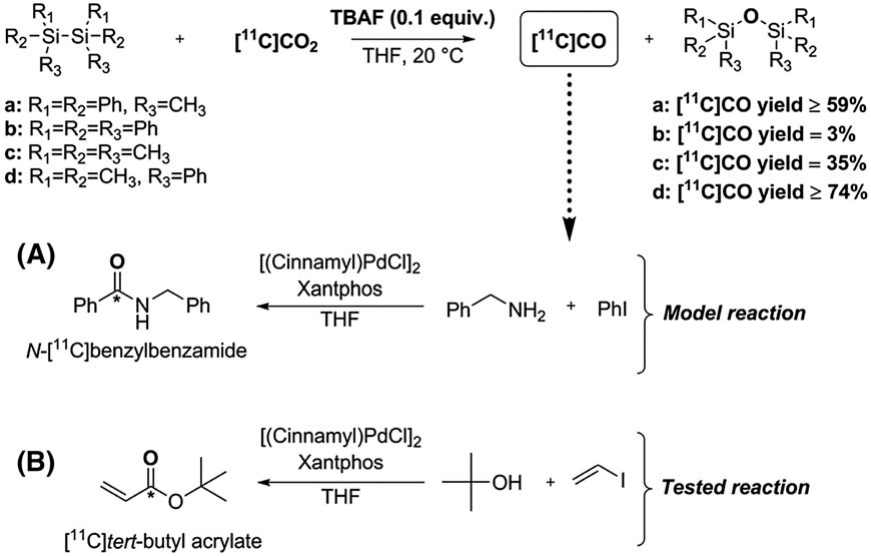
[^**11**^C]CO_2_ to [^**11**^C]CO *via* fluoride‐activated disilanes. (A) Model ^**11**^C‐carbonylation reaction; (B) tested ^**11**^C‐carbonylation reaction

The produced [^11^C]CO was used in a model ^11^C‐carbonylation reaction to yield *N‐*[^11^C]benzylbezamide in up to 74% RCY, RCP > 99%, and in an estimated A_m_ of 79 to 135 GBq/μmol^116^ within 10 minutes from EOB, Scheme 24 (A). In addition, [^11^C]*tert‐*butyl acrylate was obtained in acceptable RCY (≥ 10%) and high RCP (≥ 80%) within 10 minutes from EOB, Scheme 24 (B). This demonstrated the applicability of this [^11^C]CO synthesis process to produce different compound classes.

This [^11^C]CO_2_ to [^11^C]CO methodology utilises a simple 2‐vial labware setup and readily available reagents eliminating the remaining caveats of [^11^C]CO production *via* the [^11^C]silacarboxylic acid methodology, such as the time‐consuming pre‐synthesis reagent preparation (silyl lithium precursor) and TBAF addition post [^11^C]CO_2_ delivery.^118^

## 5 CONCLUSIONS

A broad variety of novel [^11^C]CO_2_ fixation methods are increasingly being utilised to incorporate cyclotron‐produced [^11^C]CO_2_ directly into functionalised molecules leading to a vast range of ^11^C‐compounds, such as [^11^C]amides, [^11^C]ureas, and [^11^C]carbamates. Improved synthesis loop setups have shown to enhance the rapid and efficient production of ^11^C‐tracers with minimal purification requirements and radioactivity losses. This is an important feature in routine clinical productions of PET tracers. Other [^11^C]CO fixation approaches have been introduced over recent years, such as high‐pressure apparatus, low‐pressure xenon systems, and photoinduction of the ^11^C‐carbonylation reaction. Furthermore, innovative [^11^C]CO production methodologies are emerging as alternative process to the standard oven‐based methods (Mo/Zn). In particular, the [^11^C]silacarboxylic acids to [^11^C]CO methodology and the fluoride‐activated disilanes to [^11^C]CO process may enable the low‐cost, widespread use of [^11^C]CO in diverse laboratory environments for PET tracer development without the need for specialist platforms and infrastructure.

Ultimately, this continued development and expansion of ^11^C‐chemistry will enhance the potential of PET tracer development in both clinical and research environments.

## CONFLICT OF INTEREST

None declared.
